# Early retirement among Danish female cleaners and shop assistants according to work environment characteristics and upper extremity complaints: an 11-year follow-up study

**DOI:** 10.1186/s12891-016-1053-4

**Published:** 2016-05-04

**Authors:** Lone Donbæk Jensen, Jens Peter Ellekilde Bonde, Michael Victor Christensen, Thomas Maribo

**Affiliations:** Department of Occupational and Environmental Medicine, Aarhus University Hospital, Aarhus, Denmark; Department of Occupational and Environmental Medicine, Bispebjerg-Frederiksberg University Hospital, Copenhagen, Denmark; Rehabilitation Center Marselisborg, Department of Public Health, Clinical Social Medicine and Rehabilitation Section, Aarhus University, Aarhus, Denmark; Public Health and Quality Improvement, Central Denmark Region, Aarhus, Denmark

**Keywords:** Cleaners, Follow up study, Predictors early retirement, Persistent shoulder pain, Secondary prevention

## Abstract

**Background:**

Studies have shown a negative social gradient in the incidence of early retirement. To prevent undesired early retirement, there is a need for knowledge of specific predictors in addition to social factors with a limited potential for change. The main purpose of this study was to examine musculoskeletal complaints and working conditions as predictors of early retirement among Danish female cleaners.

**Methods:**

Using Cox regression with an adjustment for extraneous factors, we compared the risk of disability pension and retirement before the nominal retirement age (65 years) in an 11-year cohort study with registry-based follow-up of 1430 female cleaners and 579 shop assistants. In subsequent analyses of female cleaners, disability pension and voluntary early retirement were modeled according to work characteristics and upper extremity complaints.

**Results:**

The adjusted hazard rate (HR) for disability pension among cleaners compared to the control group was 2.27 (95 % CI 1.58 to 3.28) and, for voluntary early retirement, 1.01 (95 % CI 0.85 to 1.20). In the subset of cleaners, the predictors of disability pension were persistent shoulder pain HR: 1.98 (95 % CI 1.47 to 2.67), elbow pain HR: 1.41 (95 % CI 1.02 to 1.94) and symptoms of nerve entrapment of the hand HR: 1.58 (95 % CI 1.14 to 2.20). Predictors of voluntary early retirement were persistent shoulder pain HR: 1.40 (95 % CI 1.16 to 1.67) and floor mopping for more than 10 h per week HR: 1.20 (95 % CI 1.03 to 1.40).

**Conclusion:**

Cleaners have a twofold higher risk of disability pension compared to the control group. Risk factors for disability pension among cleaners were persistent shoulder and elbow pain together with symptoms of nerve entrapment of the hand. The findings of specific health related predictors of early retirement could be used in secondary prevention with targeted temporary reduced workload.

## Background

Knowledge of vocational prognosis and work-related health among cleaners is sparse, although the cleaning sector employs 3.6 million people in the European Union alone [1]. There is a high occurrence of non-unionized employees in this sector, which complicates investigation [2]. A study from 2009 including measurements of exposure [3, 4] found that cleaning work was characterized by a high degree of repetition, comparable with several types of traditional unskilled occupations known from epidemiological studies to have high occurrence of upper extremities problems. Earlier studies provide some knowledge of an association of work-related diseases and professional cleaning. Contract management and limited task variation are potentially difficult to combine with less strenuous work if the cleaner has a permanent or temporary limited working capacity. Since cleaning is a labor-intensive job, competition between the cleaning companies will influence the cleaning assistants’ working conditions and work load [1]. In Denmark, disability pension and retirement before the nominal age of 65 years amounted to approximately EUR 8 billion in 2012 [5]. There are marked differences in the incidence of disability pension in different social and occupational groups [6], but the role of health and workplace factors is not fully understood [7–12]. In a follow-up study [13] comparing differences in disability pension rates in 58 industrial groups in Denmark between 1996 and 2000, the female occupational group with the highest standard incidence rate (SIR) consisted of cleaners with an SIR of 1.99 (95 % CI: 1.85 to 2.14). Other unskilled or low-educated female workers such as textile workers, meat industry workers, unskilled factory workers and nurses’ aids had significantly elevated SIRS between 1.29 and 1.44. A Norwegian follow-up study [14] found an elevated disability pension rate among cleaners compared to other unskilled occupations in a 10-year follow-up study. To prevent undesired early retirement, there is a need for knowledge about specific predictors in addition to social factors. The aim of this study was to contribute to the knowledge of risk and predictors of early retirement among cleaners by:

Comparing the cumulative incidence of disability pension and voluntary early retirement between cleaners and a control group.

Investigating the role of musculoskeletal complaints and working conditions as predictors of disability pension and voluntary early retirement among cleaners.

## Methods

### Study design

A cohort study with registry-based follow-up of risk and predictors of early retirement in a cohort of female cleaners compared with a control group of shop assistants.

### Study population and data sources

The source population was comprised of 1430 female cleaners and a reference group of 579 female shop assistants. The population to be included in the cohort was identified in registers of members hold by the 2 trade unions including name, addresses, duration of membership together with the Danish Civil Registration number (CPR). We restricted the cohort to employees above 25 years of age with more than five years of trade union membership. Persons receiving any permanent transfer income were excluded as well. The optimal control group would encompass an unskilled female occupation without physical load. Most other unskilled female occupations would have a substantial physical load as meat industry, electronic industry or nurses aids. Shop assistants with limited education was chosen as control group although we were aware there could be socioeconomic differences to the cleaners. Shop assistants who worked as cashiers were not included. Baseline data were obtained by questionnaire or telephone interview. At the follow up the CPR numbers assigned to all Danish citizens was used to link questionnaire data reliably with person-specific data from the Danish Register for the Evaluation of Marginalization (DREAM) from 1998 to 2011, inclusive. Due to major changes in the requirements for obtaining disability pension in 2012, the follow-up in the actual study ended in January 2012. Data from the DREAM database includes weekly registration of specific public transfer payments at the individual level. The original 104 different transfer payment codes from the DREAM database were coded into five variables: 1) employment, 2) sick leave, 3) unemployment benefits and other non-permanent transfer payments such as vocational rehabilitation and social assistance, 4) disability pension and 5) voluntary early retirement. Lost working years were calculated by extracting the person’s age at the year of either disability pension or voluntary early retirement from 65 years of age, which is the year for starting nominal retirement in Denmark during the years of the study. The DREAM database is thought to be close to complete, based on the economic incentive for both public and private employers to report to public authorities, and it has shown a high validity in the report of sick leave [15, 16]. There was no loss to follow-up in the two populations.

### Assessment of main outcome

The main outcomes of this follow-up study were an assessment of the risk of early retirement and predictors of early retirement among cleaners in the follow-up period. The person specific data including the two types of early retirement in the cohort was given from the DREAM register. Obtaining disability pension in Denmark requires an evaluation of work ability that has to be reduced to a minimum in any job; disability pension in this study includes flex-jobs, which were introduced in 2000 as a health-dependent, reduced-work-time scheme consistent with legislation on disability pension. Early voluntary retirement is independent of health status and available from the age of 60 if the person has been a member of an unemployment fund for 25 years, has worked for 52 week during the last three years and is available for work at the time the voluntary early retirement begins.

### Assessment of predictor variables

Information on physical and psychosocial exposure at work and complaints of pain in upper extremities were obtained from the questionnaire or interview data from the baseline investigation in 2001. Physical work factors included working hours per week and hours per week using mop systems for cleaning floors. Psychosocial work factors were measured by the short version of the Danish Copsoc inventory [17]. Health information included complaints of pain in shoulders, elbows and wrists for more than 90 days in the last year [18] and symptoms of nerve entrapment of the hand, defined as daily tingling in the fingers for the last three months.

### Assessment of covariates

Data representing poor vocational history were register-based and defined as having received more than 12 weeks of public transfer payments excluding maternity leave or educational support the year before baseline. Information about age, marital status and ethnicity was register-based as well. ‘Other ethnicity than Danish’ was defined as first- or second-generation refugees or immigrants. Educational background and BMI were self-assessed and obtained from the baseline data. Educational background was divided into primary schooling, including basic vocational courses, and further education.

### Statistical analysis

Disability pension and voluntary early retirement were analysed separately. We compared the incidence of disability pension and voluntary early retirement in cleaners and shop assistants using Cox regression with a follow-up from baseline through 572 weeks or until retirement, emigration, old age pension, or death, whichever came first. Adjustments were made for age, education level, previous vocational status and ethnicity. In subsequent analyses, disability pension and voluntary early retirement were modeled according to work characteristics and upper extremity complaints among cleaners. We did not analyze predictors of early retirement in the control group due to the limited number of persons relative to the outcome. Cox proportional hazards models were used to examine predictors of disability pension and voluntary early retirement in the follow-up period for cleaners. We evaluated the proportional hazards assumption for the exposures and the rest of the covariates. The association between each of the two outcome measures were analyzed with mutual adjustments for the full sets of predictors and covariates. The hazard ratios were estimated with 95 % confidence intervals (95 % CI).

SAS version 9.1.3 (SAS Institute Cary, NC, USA) and STATA 11.0 were used for data management and statistical analyses.

## Results

**Fig. 1:**
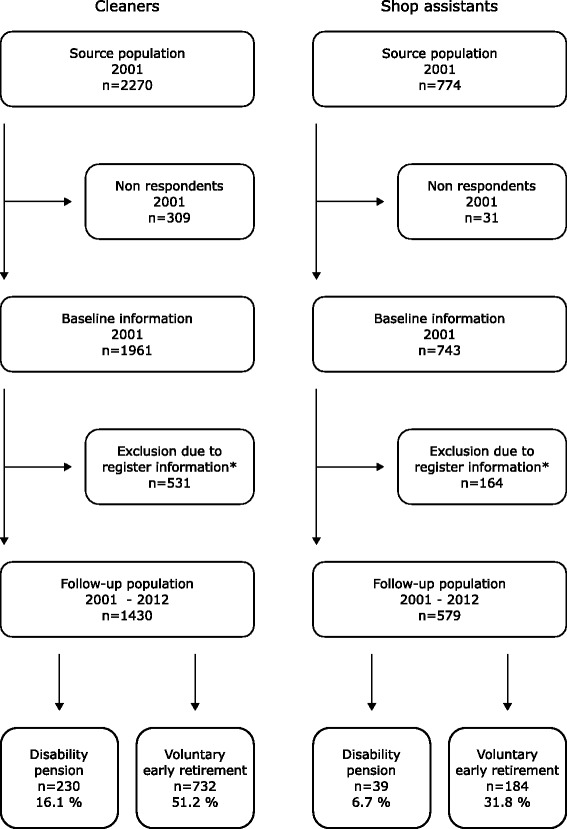
Flow chart showing the study population and numbers taking disability pension or voluntary early retirement in the follow-up period. * 531 cleaners and 164 shop assistants were excluded due to register information documenting permanent transfer income at baseline in 2001

From the baseline population, a total of 230 (16.1 %) of the cleaners and 39 (6.7 %) of the shop assistants obtained disability pension, and 732 (51.2 %) of the cleaners and 184 (31.8 %) of the shop assistants chose voluntary early retirement during the follow-up period (Fig. 1).

The mean age at disability pension among the cleaners was 51.5 years and, among the shop assistants, it was 49.4 years; the mean age at voluntary early retirement was 60.6 years for the cleaners and 61.0 for the shop assistants. In the follow-up period the cleaners altogether had 6429 lost working years due to disability pension compared to 620 in the group of shop assistants. The figures equals 4.5 lost working years per cleaner and 1.0 year per shop assistant in the follow-up period.

As can be seen from Table 1, cleaners and shop assistants differed in both age, socio-economic background, physical and psychosocial factors in addition to prevalence of symptoms in upper extremities.

**Table 1:** Baseline characteristics of cleaners and shop assistants

	Cleaners *N* = 1430	Shop assistants *N* = 579
Variable		
Age mean (sd)	50.3 (8.2)	44.0 (10.4)
Education highest level 10 years %	94.3	74.9
Less than 40 weeks paid work one year before baseline %	14.7	7.8
Other ethnicity than Danish ^a^ %	6.6	0.4
Body mass index (BMI) mean (sd)	25.6 (5.2)	24.2 (4.3)
Actual number of hours a week with paid cleaning work. Mean (sd)	28.9 (10.2)	Not relevant
Use of mop more than 10 h a week %	50.7	Not relevant
Working alone - always or often %	14.1	4.5
Decision latitude ^b^	49.1 (30.96)	60.1 (25.29)
Mean (SD) 0–100		
High value expressing high decision latitude		
Quantitative demand ^b^	45.2 (23.2)	49.0 (18.2)
Mean (SD) 0–100		
High value expressing high quantitative demand		
Shoulder pain more than 90 days in the last year %	29.9	12.6
Elbow pain more than 90 days in the last year %	16.4	6.2
Wrist pain more than 90 days the last year %	23.4	8.1
Daily tingling of fingers in the last three months %	13.4	3.6
Shoulder, elbow or wrist pain more than 90 days in the last year or daily tingling of fingers in the last three months %	45.1	19.5
Marital status % married	89.4	79.6

^a^ Information from the DREAM database; ‘other ethnicity than Danish’ means first- and second-generation refugees or immigrants

^b^ Index COPSOC [17]

**Fig. 2:**
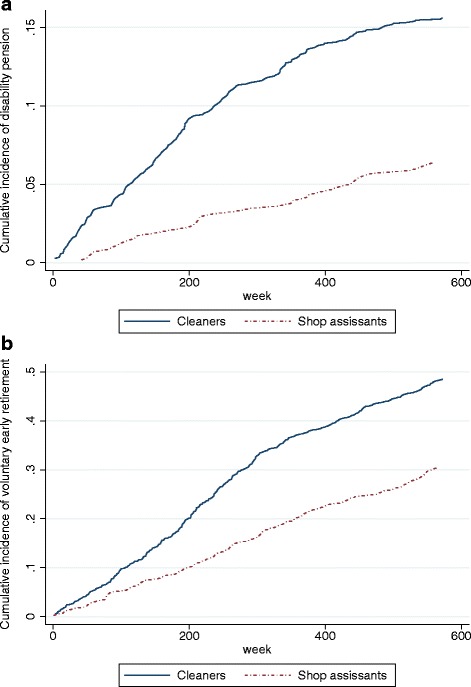
**a** Cumulative incidence curves illustrating the incidence of disability pension among the cleaners and shop assistants during follow-up. **b** Cumulative incidence curves illustrating the incidence of voluntary early retirement among the cleaners and shop assistants in the follow-up period

Figure 2a and b present the crude cumulative incidence over the follow-up period of disability pension and voluntary early retirement in the two populations.

Table 2 shows the hazard rates of taking disability pension and voluntary early retirement during the follow-up period for cleaners and shop assistants, respectively, adjusted for age, education level, previous vocational status and ethnicity.

**Table 2:** Risk of disability pension and voluntary early retirement during the follow-up period

	Shop assistants reference HR	Cleaners crude HR (95 % CI)	Cleaners adjusted ^a^HR (95 % CI)
Disability pension	1	3.16 (2.23 to 4.48)	2.3 (1.6 to 3.3)
Voluntary early retirement	1	2.18 (1.85 to 2.58)	1.0 (0.8 to 1.2)

^a^ Adjustment made for age, education level, previous vocational status and ethnicity

The reduction in risk after adjustments for the four demographic variables confirms the role of the socio-economic variables: education, vocational history and ethnicity for the risk of early retirement.

Table 3 shows predictors of taking disability pension or choosing early voluntary retirement during the follow-up period for cleaners with mutual adjustments from all variables in the model.

**Table 3:** Predictors of disability pension and voluntary early retirement for cleaners during the follow-up period

Cleaners	Disability pension HR (95 % CI)	Voluntary early retirement HR (95 % CI)
Age	1.00 (0.98 to 1.02)	1.27 (1.25 to 1.28)
Less than 40 weeks paid work the year before baseline	2.50 (1.82 to 3.43)	1.41 (1.10 to 1.82)
BMI	1.01 (0.98 to 1.03)	0.99 (0.97 to 1.01)
Use of a mop more than 10 h a week	1.01 (0.77 to 1.33)	1.20 (1.03 to 1.40)
Shoulder pain more than 90 days in the last year	1.98 (1.47 to 2.67)	1.40 (1.16 to 1.67)
Elbow pain more than 90 days in the last year	1.41 (1.02 to 1.94)	1.07 (0.85 to 1.35)
Daily tingling of fingers the last three months	1.58 (1.14 to 2.20)	0.82 (0.66 to 1.02)
High quantitative demand ^b^	0.99 (0.99 to 1.00)	1.00 (0.99 to 1.00)
Low decision latitude ^b^	1.00 (1.00 to 1.00)	1.00 (1.00 to 1.00)
Other ethnicity ^a^	1.46 (0.97 to 2.19)	0.80 (0.46 to 1.12)

^a^ Information from the DREAM database; ‘other ethnicity than Danish’ is comprised of first and second generation of refugees or immigrants

^b^ Index COPSOC [17]

The two types of early retirement shared the predictors of poor vocational history and shoulder pain more than 90 days in the last year. Two other upper extremities complaints – elbow pain more than 90 days in the last year and symptoms of nerve entrapment – also predicted disability pension. In addition, floor cleaning defined as use of a mop more than 10 h a week was associated with risk of early retirement. The high quantitative demand and low decision latitude of psychosocial working factors were not associated with either of the types of early retirement.

## Discussion

In this occupational cohort of 1430 female cleaners, a total of 66 % retired earlier than the normal age of pension during the 11-year follow-up period. The cleaners had an adjusted risk of more than twofold obtaining disability pension compared to the control group. There was no difference in the adjusted risk of voluntary early retirement between the two groups. The findings are consistent with results from previous studies showing that cleaners have a substantially higher risk of disability pension than females in other unskilled occupations [12, 13]. Previous attachment to the labor market and serious shoulder complaints predicted both disability pension and voluntary early retirement. Long-lasting shoulder pain can be a serious condition with a high surgery rate [19] for which it is most likely that a long course of therapy with treatment-resistant symptoms could lead to a permanent disabling condition. It was, however, unexpected that elbow symptoms and symptoms of nerve compression of the hand would predict disability pension since those diagnoses do not usually fulfill the criteria for obtaining disability pension in Denmark. More than 10 h of floor mopping per week predicted voluntary early retirement, which could be explained by the difficulty of working as a cleaner if you have persistent shoulder pain because repetitive tasks and somewhat forceful shoulder movements constitute a substantial part of the working day. The design of the study with exposure and symptoms measured at the same point does not allow conclusions about whether floor work causes shoulder symptoms. We did not find studies exploring a broader range of possible predictors of early retirement among cleaners. Compared to a previous Danish study among nurses’ aides (measuring vocational prognosis) similar to the present study [20], we found comparable findings in predictors of disability pension. The role of musculoskeletal disorders as predictors of disability pension is in accordance with a large Swedish nationwide cohort study in which people on sick leave in 2005 due to musculoskeletal diagnoses had a considerably increased risk of all causes of disability pension in the following three years compared to those on sick leave for other causes [10]. BMI in the present study did not predict disability pension, contrasting with results from a present review [21]. A strength of the present study is its prospective design with a long follow-up in an almost complete and time-accurate national registry with no loss to follow-up in the target population. The registry-based information on sick leave, unemployment and other types of transfer income the year before baseline is a valuable tool to detect socioeconomic residual confounding. We choose to use persistent symptoms from the category >90 days of musculoskeletal complaints in the last year from the Nordic questionnaire to make sure the symptoms had a relevant health impact together with the notion that long-lasting symptoms are less prone to contribute to information bias. Exposure data and symptoms are self-reported and measured at one point only; this means that exposure and symptoms may interact and that there is a possibility that exposure and symptoms may change during follow-up. We have information on change in unemployment fund during follow-up in which 16 % changed to another unemployment fund. 64 % of those changed to another unskilled occupation, leaving 5.5 % of the population unaccounted for. The study design does not allow us to conclude if the increased risk observed in cleaners was caused by their job The control group established at baseline differed in several aspects. At the planning of the cross-sectional study, we were aware of the difficulties in finding a suitable control group without exposure to strain on upper extremities, and shop assistants were thought to be the best choice. The possibility for adjustment and especially the knowledge of the social history before baseline are thought to reduce the problems of the marked differences between the two groups at baseline. Information on health issues is limited to symptoms of pain in upper extremities. The study included both public and private workplaces in which there have been changes towards more intensive and repetitive work due to increased competition in cleaning contracts in both sectors. With respect to generalization to other countries, both differences in legalization across countries and differences in working conditions have to be taken into account.

## Conclusion

This study supports the view that cleaners have a substantially higher risk of receiving disability pension than a control group of shop assistants. We have no information about the exact health-related causes of disability pension, but given that experiencing shoulder pain for more than 90 days over the course of the last year before baseline predicted both disability pension and voluntary early retirement, it would appear to be an important target for secondary prevention of early retirement in the cleaning profession.

## Ethics approval

Danish data protection agency 2009-41-3431.

## Availability

The data analyzed in the study is a part of a Danish musculoskeletal database where access to data is possible after approval by the committee responsible of the database.

## Abbreviations

BMI: body mass index
COPSOC: The Copenhagen Psychosocial Questionnaire
CPR: Danish civil registration number
DREAM: the Danish Register for the Evaluation of Marginalisation
EMG: electromyography
